# Efficacy and Safety Evaluation of a Ready-to-Use Bivalent Porcine Circovirus Type 2 and *Mycoplasma hyopneumoniae* Vaccine in China

**DOI:** 10.3390/vaccines13121203

**Published:** 2025-11-28

**Authors:** Huimeng Yan, Yupeng Mo, Wanfa Luo, Xiong Xie, Zeyu Li, Shuming Tang, Xiaoxin Liu, Qi Cao, Hongyao Lin, Di Gao, Ruben Del Pozo Sacristan, Xiaoye Wang

**Affiliations:** 1 College of Animal Science and Technology, Guangxi University, No. 100, University Road, Xixiangtang District, Nanning 530004, China; hui.meng.yan@msd.com (H.Y.);; 2 MSD Animal Health (Shanghai) Trading Co., Ltd., 1582 Gumei Road, Xuhui District, Shanghai 200233, China; 3 Global Statistics & Data Management, MSD Animal Health, Wim de Körverstraat 35, 5831 AN Boxmeer, The Netherlands; 4 MSD Singapore MSD Animal Health Innovation Pte Ltd., 1 Perahu Road, Singapore 718847, Singapore; hongyao.lin@msd.com

**Keywords:** *Mycoplasma hyopneumoniae*, porcine circovirus type 2, vaccination, swine, bivalent vaccine, sustainability

## Abstract

**Background/Objectives:** Porcine Circovirus Type 2 (PCV2) and *Mycoplasma hyopneumoniae* are primary pathogens causing respiratory disease in pigs. Recently, a Ready-to-Use bivalent PCV2 and *M. hyopneumoniae* vaccine has been registered in China. The aim of this randomized, side-by-side trial was to evaluate the efficacy and safety of this vaccine under field conditions in a Chinese commercial pig farm. **Methods:** In total, 938 piglets were allocated to three groups—A (tested vaccine), B, C—and vaccinated according to different schemes. Efficacy was assessed by Average Daily Gain (ADG), pneumonia lesions at slaughter and PCV2 viremia. Systemic reactions were recorded after vaccination to evaluate safety. **Results:** ADG was higher in group A compared with other vaccination schemes. The prevalence of pneumonia lesions was significantly lower in group A. PCV2 viremia was overall low in all groups, with no reported differences. No severe or moderate systemic reactions were observed after vaccination. Only four pigs showed mild reactions (A: 2/320, B: 2/309; C: 0/309). **Conclusions:** Under these conditions, the tested vaccine was proved to be efficacious in increasing ADG and reducing pneumonia at slaughter by protecting against both PCV2 and *M. hyopneumoniae* field infections. It can also be concluded that the Ready-To-Use bivalent vaccine was safe.

## 1. Introduction

Porcine Circovirus type 2 (PCV2) and *Mycoplasma hyopneumoniae* are primary pathogens causing respiratory disease in pigs and play a key role in the Porcine Respiratory Disease Complex [1]. Porcine Circovirus Disease (PCVD) is caused by PCV2 and may have different clinical manifestations, including systemic and reproductive disease, subclinical infection and porcine dermatitis and nephropathy syndrome [2]. PCV2-respiratory disease is considered as an entity within PCV2-systemic disease and its mainly characterized by respiratory distress, dyspnea and proliferative and necrotizing pneumonia [3]. Since the introduction of commercial vaccines, the clinical and economic impact of PCV2 infections has decreased [4]. As a multifactorial disease, PCVD control not only depends on vaccines but also relies on the prevention or elimination of other triggering elements, such co-infections and environmental factors [5]. *M. hyopneumoniae* is the etiologic agent of Enzootic Pneumonia, a chronic respiratory disease characterized by coughing, insidious bronchopneumonia and cranioventral pulmonary consolidation [6]. *M. hyopneumoniae* colonizes the cilia of the respiratory epithelium in the airways, leading to a suppression of both innate and acquired local immunity, which facilitates colonization by commensal and secondary pathogens and increases the severity of the respiratory disease [7]. Enzootic pneumonia has a negative economic impact on the pig industry, mainly due to reduced performance, uneven growth, longer time to reach slaughter weight and costs associated with treatment and control [8]. Control of enzootic pneumonia can be achieved by antimicrobial therapy, vaccination and optimization of management and housing conditions [6].

In practice, pig herds affected by single-pathogen infections are uncommon, whereas most of the commercial farms are co-infected with multiple microorganisms. Combined infection of pigs with PCV2 and *M. hyopneumoniae* results in more severe respiratory disease [1], as both predispose pigs to infections with secondary pathogens [4,8]. Both agents have the potential to reduce farm efficiency by decreasing growth performance, increasing mortality rates, and causing more extensive and severe lung lesions [4,6,9]. This reduced performance negatively impacts the economic sustainability of pig herds [4,6].

First isolation of PCV2 in China was in 2000 [10], and since 2009 several commercial vaccines have been registered in the country. Despite its wide use in the field [11], PCV2 still causes major losses to the pig industry [12,13,14]. This situation has prompted increased attention to PCV2 by Chinese producers and authorities in recent years [15,16] and had motivated support from the government to advance research on molecular epidemiology and vaccine development [15]. Likewise, *M. hyopneumoniae* is highly prevalent across the country and producers continuously faces challenges in controlling the disease [17,18].

By the end of 2018, the Chinese swine industry was hit by African Swine Fever Virus [19]. This has led to a profound restructuring of the pig sector, that shifted from mostly family backyard production to large-scale production, where efficiency and cost savings are key to success [20]. Therefore, the need for a successful and sustainable control of both PCVD and Enzootic Pneumonia is now greater than ever.

Recently, a Ready-to-Use (RTU) bivalent PCV2 and *M hyopneumoniae* vaccine has been registered in China (Porcilis^®^ PCV M Hyo; MSD Animal Health, Rahway, NJ, USA). Previous research has demonstrated excellent efficacy and safety under a variety of production systems in Europe [21,22,23,24,25]. However, the performance of this vaccine under Chinese conditions and its potential economic profitability remains unexplored.

In this study, the performance of an RTU bivalent PCV2 and *M. hyopneumoniae* vaccine was investigated in a commercial pig herd in China. A field trial was conducted to evaluate vaccine efficacy and safety. In addition, an economic assessment was carried out to estimate the benefits of such strategy. Preliminary results showed that the tested vaccine was efficacious and safe under the investigated field conditions, and revealed an economic benefit, providing a practical basis for further evaluation of vaccines impact on sustainability.

## 2. Materials and Methods

### 2.1. Herd Selection

A pig herd with history of PCV2 and *M. hyopneumoniae* infections was selected for this study. Prior to the initiation of the study, a serological investigation was performed to evaluate the health status. Sows, nursery pigs and fatteners were sampled. All blood samples tested negative by Polymerase Chain Reaction (PCR) for Porcine Reproductive and Respiratory Syndrome virus (PRRSV) and PCV2. Most of the sows (80%), 20% of nursery piglets and 53% of fatteners had antibodies against *Actinobacillus pleuropneumonaie*. Oral swabbing of suckling piglets revealed a stable situation for *M. hyopneumoniae*: 3 out of 30 piglets tested PCR positive at weaning.

The herd was a wean-to-finish farm (site 2) located in Guanxi Province (China), with a housing capacity for 1000 pigs, operating in an all-in, all-out system and receiving piglets weaned at 21 days of age. Piglets were sourced from a breeding unit (site 1; 1800 sows) with a commercial health status (PRRSV-negative; Pseudorabies virus-negative; *M. hyopneumoniae*-positive, PCV2-positive). Prior to weaning, piglets were routinely vaccinated against PCV2 and *M. hyopneumoniae.* During the nursery period, routine antimicrobial medication was administered to the piglets (amoxicillin at 4 and 7 weeks of age). Fatteners were dewormed (doramectin) at starting of the fattening period.

### 2.2. Experimental Design

A randomized, parallel-group trial was performed in the aforementioned wean-to-finish pig farm. In total, 938 piglets aged 21 days were selected in the breeding unit on day of weaning.

After weaning, piglets were transported to the trial wean-to-finish farm, individually tagged (LeeO^®^ tag, Extra RFID, Beijing, China), weighed (LeeO^®^ scale, LeeO Precision Farming B.V, Deventer, The Netherlands), and randomly allocated to three groups (A, B, C) using LeeO^®^ Software V2025.10.4 (LeeO Precision Farming B.V, Deventer, The Netherlands). Thereafter, pigs were intramuscularly vaccinated against PCV2 and *M. hyopneumoniae* according to the manufacturers’ specifications, following the schemes below:

- Group A (experimental group): 320 piglets intramuscularly vaccinated in the neck at 21 days of age with Porcilis^®^ PCV M Hyo (2 mL) (MSD Animal Health, Rahway, NJ, USA).
- Group B: 309 piglets intramuscularly vaccinated in the neck at 21 days of age with Ingelvac CircoFLEX^®^ (1 mL) (Boehringer-Ingelheim, Ingelheim am Rhein, Germany) mixed with Ingelvac MycoFLEX^®^ (1 mL) (Boehringer Ingelheim, Ingelheim am Rhein, Germany).
- Group C: 309 piglets intramuscularly vaccinated at 21 days of age in each side of the neck with Ingelvac CircoFLEX^®^ (1 mL) (Boehringer Ingelheim, Ingelheim am Rhein, Germany) and Suvaxyn M Hyo^®^ (Zoetis Inc., Parsippany, NJ, USA) (2 mL), followed by a second dose of Suvaxyn M Hyo^®^ (Zoetis Inc., Parsippany, NJ, USA) (2 mL) at 42 days of age.

Ethical constraints precluded the inclusion of a control group. The vaccine investigated in this study (Experimental group; Porcilis^®^ PCV M Hyo) is a Ready-to-Use (RTU) bivalent vaccine containing baculovirus-expressed ORF2 antigen of PCV2 and inactivated *M. hyopneumoniae* cells. Both antigens are dissolved in an adjuvant based on a combination of an oil-in-water emulsion with aluminum hydroxide (Emunade^®^, MSD Animal Health, Rahway, NJ, USA). The vaccine was administered intramuscularly to three-week-old piglets as a single 2 mL dose according to the manufacturer’s recommendations.

Immediately following vaccination, pigs were housed in slatted pens (20 pigs per pen) according to the vaccination group. Pigs were fed standard commercial diets from weaning through slaughter, providing sufficient nutrient levels to satisfy minimum requirements and support normal growth performance. Pigs always had ad libitum access to feed and water.

### 2.3. Efficacy Assessment

To assess the efficacy of the vaccines under field conditions, three main parameters were used: Average Daily Gain (ADG) reflecting the control of both pathogens (PCV2 and *M. hyopneumoniae*); Lung Lesion Score (LLS) at slaughter for prevalence and severity of Mycoplasma-like lesions; and PCV2 viremia (genomic serum load) for protection against PCV2 infection. Other clinical parameters were also recorded and considered secondary.

#### 2.3.1. Performance

Growth performance was assessed by weighing individual pigs at weaning/vaccination (day 0), at transfer from growing to fattening (day 100), and prior to slaughter. LeeO^®^ scale (LeeO Precision Farming B.V, Deventer, The Netherlands) was used to weigh and record live weight of weaners at day 0, whereas a day 100 and prior to slaughter a commercial scale was equipped with a LeeO^®^ head (BWT Weegtechniek NV, Boxtel, The Netherlands).

Average Daily Gain (ADG) was assessed for three periods: nursery-growing period (weaning or day 0 to day 100), finishing period (day 100 to slaughter), and overall period (wean-to-slaughter). Days to reach slaughter weight were recorded.

Mortality was documented during the whole study. Percentage of pigs below the slaughter weight (<70 kg) at the end of the study (runt %) was also recorded. Both mortality % and runt % were considered secondary parameters.

#### 2.3.2. Lung Lesion Evaluation at Slaughter

At slaughter, the prevalence and the severity of lung lesions, both bronchopneumonia and pleuritis, were evaluated. Bronchopneumonia lesions or Mycoplasma-like lesions were defined as purple-to-gray lesions of pulmonary consolidation, mainly affecting the cranioventral parts of the lungs, with unilateral or bilateral distribution [26]. For the evaluation of bronchopneumonia lesions, the scoring proposed by Madec and Kobisch [27] was used. First, each pulmonary lobe was scored from 0 to 4 points according to the percentage of area affected (0: no lesion; 1: <25%; 2: 25% to <50%; 3: 50% to <75%; 4: 75% to 100%). After that, the final individual score for each lung was calculated as the sum of all lobe scores. And finally, the average LLS, which indicates the severity of pneumonia lesions, was calculated as the mean of all individual lung scores for each treatment group. The prevalence and severity of lesions compatible with *Actinobacillus pleuropneumoniae* infections, mainly pleuropneumonia, pleuritis, and/or hemorrhagic, necrotic or caseous abscesses, were investigated using the Slaughterhouse Pleurisy Evaluation System (SPES) method [28].

#### 2.3.3. Sera Collection

A longitudinal blood sampling was performed during the study. In total, 25 pigs per group and age category were bled at 7, 11, 15, 19, 23, and 25 weeks of age. Serum samples were frozen at −80 °C until the following lab analyses were performed:

- PCV2 viremia
  Each serum sample (*n* = 25/group) was analyzed for the detection of PCV2 and quantification of the viral load using a real-time PCR test (Porcine Circovirus Type 2 Quantitative Kit^®^, Tianjin Odrei Biomedical Technology Co., Ltd., Tianjin Shi, China). The lower limit of detection of this kit is 3 target copies/µL. Area under the curve (AUC) was calculated by the linear trapezoidal rule as a measure of total shedding over time.
- Serological investigation
  In total, 10 serum samples (per group and age category) were investigated for the presence of antibodies against PCV2 (Porcine Circovirus Type 2-d Cap-ELISA Antibody Test Kit, Beijing Jinnuo Baitai Biotechnology Co., Ltd., Kino, Republic of Korea), *M. hyopneumoniae* (IDEXX *Mycoplasma hyopneumoniae* Antibody Test Kit, Beijing IDEXX Biotechnology Co., Ltd., Beijing, China), and *A. pleuropneumoniae* (IDEXX *Actinobacillus pleuropneumoniae* Antibody Test Kit, Beijing IDEXX Biotechnology Co., Ltd., Beijing, China). Results were expressed as negative, positive, or doubtful according to the manufacturer’s recommendations.

#### 2.3.4. Laryngeal Swab Collection

A longitudinal laryngeal sampling was performed during the study. Pigs were swabbed at 11, 15, 19, 23, and 25 weeks of age. In total, 25 pigs/group were sampled at each age category, except at 7 weeks (*n* = 10 pigs/group). Swabs samples were analyzed by real-time PCR (*Mycoplasma hyopneumoniae* Quantitative Kit^®^, Tianjin Odrei Biomedical Technology Co., Ltd., Tianjin, China).

### 2.4. Safety Assessment

The general health of the piglets was checked at admission and only healthy piglets were enrolled in the study. Safety was evaluated by recording severe, moderate, and mild systemic reactions at the time of vaccination, 4 h and 24 h after vaccination and then at 4, 7 and 14 days post-vaccination. Animals were observed daily at group level until the end of the study. In addition, the presence of local reactions at the site of injection was evaluated at 1, 7 and 14 days post-vaccination, by recording redness, soft/hard swelling, abscesses and/or absence of local response. Rectal temperature was not measured due to concerns with African Swine Fever.

### 2.5. Economic Assessment

An economic assessment was performed using the differences in performance parameters and lung lesions among the vaccination groups. Revenue, costs and net benefit were calculated to estimate the improvement in farm income among groups, using the results of Group A (tested vaccine) as the reference for comparison. The following values were used for this calculation: the average price per kilogram of a live pig sold at the abattoir (RMB 15.09/Kg live weight [29]; average price of a healthy lung sold at the abattoir (RMB 2.00/lung); price of a needle (RMB 1.00/unit; manufacturer estimate). The cost of needle per vaccinated pig was adjusted to the cost per pig sold. Similarly, data was corrected taking into consideration the mortality percentage for each group (RMB 9.68/pig placed for each incremental improvement of 1%) [30].

### 2.6. Statistical Analysis

The number of 304 animals in each treatment group permitted to assess a difference of 20 g (standard deviation = 88) in ADG with 95% confidence and 80% statistical power. The pig was the statistical unit. Differences were considered as statistically significant when *p*-values were lower than 0.05 (two-sided test). Statistical analyses were performed using SAS^®^ software 9.4 (SAS Institute Inc., Cary, NC, USA).

Main parameters for analysis were ADG and Lung Lesion Score. Both, live weight at slaughter and ADG during all three periods, were analyzed by AN(C)OVA model with treatment group as a fixed effect and initial weight as a covariate and using Tukey’s post hoc test to compare groups. Lung Lesion Score for both pneumonia and pleuritis, were compared among groups using Wilcoxon rank-sum test. The proportion of lungs with pneumonia lesions, the proportion of pigs with pleuritis lesions, as well as mortality % and runt % were compared among groups by Fisher’s exact test.

### 2.7. Ethical Statement for Experimental Procedures

The animal experimental procedure was reviewed and approved by the ethical committee of Guangxi University, with approval number GXU-2025-350.

## 3. Results

### 3.1. Efficacy Assessment

#### 3.1.1. Performance

Weight at weaning (day 0) was homogeneous at baseline among all groups (Table 1). Live weight prior to slaughter was significantly higher in pigs from Group A compared to B (by 4.0 kg) and C (by 4.2 kg) (*p* < 0.05) (Table 1).

**Figure 1:**
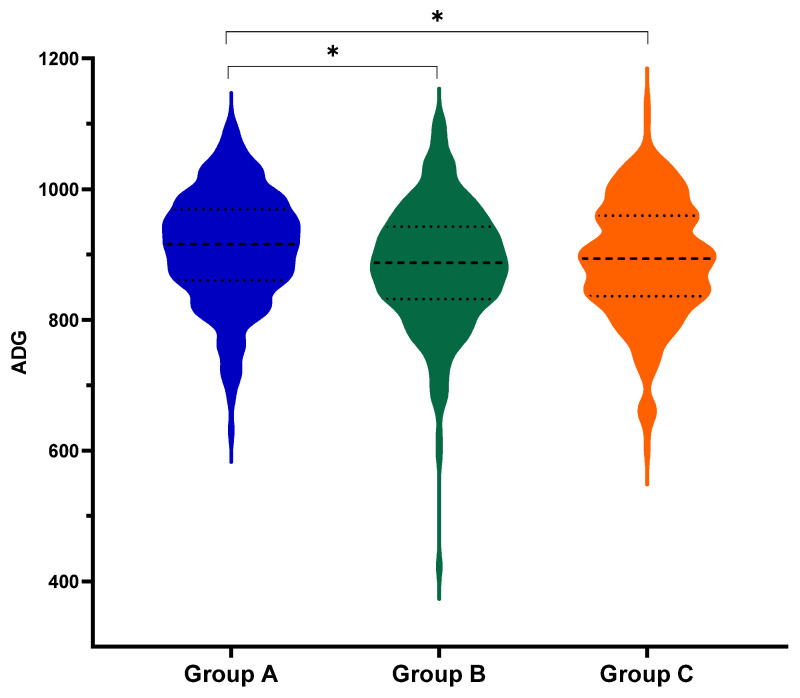
Performance results. Spread of Average Daily Gain during the overall period in pigs vaccinated with three different schemes (A, B and C). Coefficient of variation of ADG in Group A (9.14%), B (9.83%) and C (9.97%). * shows significant differences between groups (*p* < 0.05). Dashed (----) and dotted (….) lines refer to median and quartiles, respectively.

Average Daily Gain was significantly improved in pigs from Group A, compared with the other groups during the nursery-growing phase (*p* < 0.05), and during the overall period (weaning to slaughter) (*p* < 0.05) (Table 1). In addition, the spread of live weights prior to slaughter was the lowest in Group A (8.71%), as shown by the coefficient of variation (Table 1). Similarly, more homogeneous growth was observed in group A (9.14%), as described by the coefficient of variation for the ADG during the overall period, when compared with group B (9.83%) and C (9.97%) (Figure 1).

Mortality percentage was not different among groups (*p* > 0.05). In total, 49 pigs died during the study (A: 17/320; B: 14/309; C: 18/309). Different causes of death were reported, mostly associated with digestive signs (34.7%), central nervous signs (28.6%), respiratory signs (14.3%), sudden death (12.2%), and others (10.2%). In most of the cases associated with digestive signs, either PEDV or bowel syndrome was diagnosed. *Streptococcus suis* or *Glaesserella parasuis* were also isolated in several of the cases associated with nervous signs or sudden death. *G. parasuis* was also cultured in two cases with respiratory signs. The number of pigs below the slaughter weight (<70 kg) by the end of the study was low (A: 2/320; B:2/309; C: 0/309) (*p* > 0.05) (Table 1). None of the dead or culled pigs showed signs of PCVD, namely wasting, poor growth and/or weight loss.

#### 3.1.2. Lung Lesions at Slaughter

Prevalence of pneumonia (*p* < 0.05) was significantly reduced in group A compared with groups B and C (Table 2). Severity of pneumonia (LLS) was not different between group A and C (*p* > 0.05), but significantly lower in both groups compared to group C (*p* < 0.05). Prevalence of pleuritis was not different among all three groups (A: 4.8%; B: 3.0%; C: 3.1%) (*p* > 0.05).

#### 3.1.3. Sera Sampling

**Figure 2:**
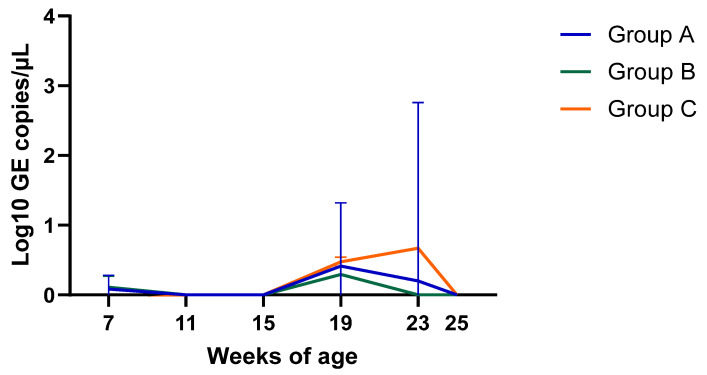
Time-course of PCV2 viremia as determined by qPCR. Results were expressed in Log10 GE copies/µL. No significant differences were observed among groups.

**Figure 3:**
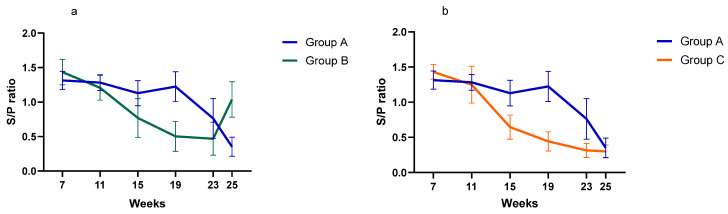
Time-course of anti-PCV2 antibody development in vaccinated pigs from Groups A and B (**a**) and Groups A and C (**b**). Bars indicate 95% confidence interval. Results were expressed in S/P ratio (S/P < 0.4 is considered negative; S/P > 0.4 is considered positive).

**Figure 4:**
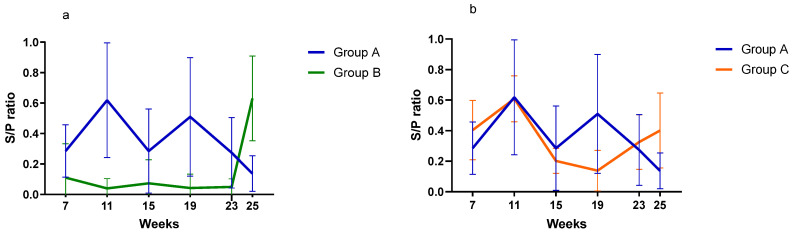
Time-course of anti-*M. hyopneumoniae* antibody development in vaccinated pigs from Groups A and B (**a**) and Groups A and C (**b**). Bars indicate 95% confidence interval. Results were expressed in S/P ratio (S/P < 0.3 is considered negative; S/P 0.3–0.4 is considered doubtful; S/P > 0.4 is considered positive).

- PCV2 viremia
  PCV2 viremia remained either undetectable or at very low levels during the whole study in all three groups. Time course of PCV2 viremia in is plotted in Figure 2; the average Log10 GE copies/µL shown is limited to the few viremic pigs. Peak of viremia occurred between 19 and 23 weeks of age, and those viremic pigs had very low viral load (<0.7 Log10 GE copies/µL). Due to this low viremia, sequencing was unsuccessful, and the genotype was not determined. The AUC of all sampled pigs was 2.54, 0.71, and 1.74 (*p* > 0.05) for Group A, B, and C, respectively.
- Serological investigation
  Results from the serology investigation are displayed on Figure 3 and Figure 4, and Table 3. Overall, PCV2 antibodies were present in all three groups at 7 weeks of age. In Group A, average S/P ratio remained stable up to 19 weeks and then declined. For Groups B and C, the decline was much more pronounced from 11 weeks onward. Pigs from Group B seroconverted at 25 weeks (90% seropositive).
  Regarding *M. hyopneumoniae*, the serology investigation revealed different infection dynamics among groups (Figure 4). In Group A, S/P ratio values and seroprevalence (60%) peak at 11 weeks, and then gradually declined from 19 weeks of age onward. For Group B, most pigs remained seronegative during the study, except for a large seroconversion (80%) at 25 weeks. All pigs from Group C had seroconverted (100%) by 11 weeks, then S/P values and seroprevalence declined to a minimum, to slightly increase again after 19 weeks. For *A. pleuropneumoniae*, no differences were observed among groups (Table 3).

#### 3.1.4. Laryngeal Swab Collection

All samples were *M. hyopneumoniae* PCR-negative at 7, 11, and 15 weeks. At 19 weeks, one sample each from Groups B and C was PCR-positive. Group A remained negative at 19 weeks but had one positive sample at 23 weeks. At 25 weeks, all samples in all groups were again PCR-negative. The Ct values of the positive samples were 36–37.

### 3.2. Safety Assessment

No severe or moderate systemic reactions were described after vaccination. Only four pigs (A: 2/320, 0.62%; B: 2/309, 0.65%) presented mild reactions, characterized by animals lying down with decreased activity and rapid breathing. All affected animals fully recovered within 15 min after vaccination (Table 4). No local reactions at the injection site were detected, neither local abscess.

### 3.3. Economic Assessment

The net profit of the pigs vaccinated in Group A was RMB +54.20/pig sold and RMB +69.30/pig sold compared with pigs from Groups B and C, respectively (Table 5).

## 4. Discussion

In the present study, the efficacy and safety of an RTU bivalent PCV2 and *M. hyopneumoniae* vaccine recently registered in China were investigated under commercial field conditions. The tested vaccine improved the growth performance and reduced the prevalence of lung lesions when compared with other vaccination schemes. PCV2 viral load in serum remained either undetectable or at very low levels during the study. The safety profile of the investigated vaccine was similar to that of the other vaccination protocols. Additionally, an economic benefit was demonstrated.

The current situation in the Chinese swine industry motivates the search for alternative solutions that ensure sustainable food production while maintaining high health and welfare standards, using fewer resources, and being economically profitable. Control of both PCV2 and *M. hyopneumoniae* infections is key to overcoming these industry challenges.

PCV2 was first identified in North America [31] and Europe [32] in 1998. The first isolation in China dates from 2000 [10], and the first commercial PCV2 vaccines were available in the country in 2009. Most of the PCV2 vaccines registered in the Chinese market today are inactivated (70%), while subunit vaccines are less common [15,16,33]. Generally, both types of PCV2 vaccines are considered highly efficacious against PCV2 infection [34]. However, the cellular immunity stimulated by subunit vaccines is often stronger, while in inactivated vaccines, it is less pronounced and often requires adjuvants to enhance it [16]. Despite PCV2 vaccination being the first control strategy adopted by producers worldwide and the wide PCV2 vaccine penetration in the Chinese pig industry (77%) [11], the virus is still having a negative impact on this industry [12,13,14]. Since July 2017, the Ministry of Agriculture of China has promulgated a policy that promotes the sustainable development of large-scale farming [12]. As a consequence, a shift in the epidemiology of PCV2 has been suggested at the country level [12,13,14]. A meta-analysis performed by Liu et al., 2020 [12], confirmed that PCV2 was still very prevalent at the country level in 2017 (51.9%; 95% CI: 32.1–71.8). This study also revealed that PCV2 was more prevalent in intensive (50.1%) than in extensive farms (37.5%) [12]. This situation has motivated the increased attention given to PCV2 by Chinese producers and authorities in recent years [15,16].

*M. hyopneumoniae* was isolated for the first time in China in 1973 [17]. The first commercial vaccines were available in the early 1990s. Since then, many vaccines have been registered, most of which are inactivated [33]. Despite the broad availability of vaccines against *M. hyopneumoniae*, vaccine uptake in the pig industry remains far from ideal. This may explain the high prevalence of *M. hyopneumoniae* infections reported [17]. The latter study investigated clinical samples collected between 2018 and 2020 in 215 herds from 27 provinces in China by using nested PCR. It described individual positive rates of 7%, 18% and 44% for samples collected in 2018, 2019, and 2020, respectively; while within-herd prevalence was 14%, 54% and 56% for the same periods [17].

All the above confirms that pig producers continue to suffer a high burden of PCV2 and *M. hyopneumoniae* infections and motivates the search for new efficacious and sustainable alternatives. This justifies the recent registration of a new RTU bivalent PCV2 and *M. hyopneumoniae* vaccine. As a bivalent vaccine, it includes a PCV2 ORF2 subunit consisting of Virus-Like Particles (VLPs) and an inactivated *M. hyopneumoniae* strain [21]. At present, commercial PCV2 vaccines provide strong clinical and virological protection [2,16]. For subunit vaccines, VLPs mimic the viral structure and stimulate a strong B- and T-lymphocyte-mediated response, inducing specific antibody production [16]. This supports the large-scale production of the PCV2 antigen.

The study was designed to evaluate the performance of the tested vaccine from an efficacy, safety, and economic perspective. Due to ethical reasons, no control group was included, which means that all three groups were vaccinated against PCV2 and *M. hyopneumoniae*. Therefore, the tested vaccine was compared with two vaccination scenarios commonly found in practice, which differ in convenience and labor. In the case of Group C, pigs were vaccinated three times, receiving one dose of PCV2 vaccine and two doses of *M. hyopneumoniae* vaccine with three week’s interval between doses. Therefore, those pigs were handled twice and received three intramuscular injections, which increases stress and the risk of iatrogenic infections [35,36,37,38]. Group B received a single injection, but prior to administration, the vaccine required preparation and mixing. Pigs from group A were injected only once with an RTU vaccine that did not require preparation, thus offering the highest level of user convenience and requiring the least labor [39].

The evaluation of vaccine efficacy for PCV2 and *M. hyopneumoniae* vaccines is challenging, especially under field conditions. Although there is no universal standard for PCV2 vaccines [16], most studies include quantification of viral load in blood or tissues, scoring of pathological lesions, measurement of PCV2-specific antibodies, assessment of cellular immune response, and evaluation of growth performance. Regarding *M. hyopneumoniae*, efficacy studies focus on recording clinical signs, measuring growth performance, and assessing macroscopic Mycoplasma-like lesions and microscopic histological changes [40]. In our clinical trial, performance, pneumonia lesions at the abattoir and PCV2 viremia were the main parameters studied. Vaccination against PCV2 and *M. hyopneumoniae* has other benefits that have not been investigated in this trial. Potential effects of long-term vaccine implementation include the enhancement of herd immunity, the reduction of antimicrobial usage and the improvement of welfare standards due to fewer handling events.

Growth performance evaluation indicated that the vaccination scheme used in Group A was associated with improved growth compared with the other schemes. Pigs in Group A achieved an ADG > 900 g/day from weaning to slaughter, a higher average live weight at slaughter (+4 kg), and an ADG increase of 20–26 g/day, along with narrower dispersion in ADG and slaughter weight. Uneven growth is a common finding in *M. hyopneumoniae* infections [8] and has multiple economic and management consequences. However, the literature rarely addresses the toll of this growth variation. Infected animals frequently exhibit poorer feed efficiency and slower weight gain, resulting in a group-wide discrepancy between nutrient intake and growth demands. This variability complicates feeding strategies and may increase feed costs. Additionally, contracted farms face challenges in maintaining consistent fattening cycles, as pigs require extended periods to reach market weight, reducing turnover rates and overall productivity. At the abattoir, pigs outside the targeted weight range may incur financial penalties, further impacting profitability.

The assessment of lung lesions at the abattoir confirmed that the newly registered vaccine kept enzootic pneumonia under control. It is important to note that, although cranioventral pulmonary consolidation is a characteristic lesion observed in cases of enzootic pneumonia, these bronchopneumonia lesions are not pathognomonic for *M. hyopneumoniae* infections [26]. Therefore, laboratory and histopathological analysis of lung tissue would have helped to confirm the etiology of those lesions. Nevertheless, the reduction in prevalence of these pneumonia lesions in the experimental group, compared with the other two vaccination schemes, and the different *M. hyopneumoniae* infection dynamics among groups, confirmed the ability of the tested vaccine to control enzootic pneumonia. Pigs from Groups A and C seroconverted after vaccination (7–11 weeks of age), whereas no seroconversion was detected in group B. This seroconversion in two groups and the absence of antibodies in a third one suggest that those were not maternal-derived antibodies, since all piglets were randomized at the start of the trial to ensure an equal distribution across groups. While the reason for the 100% seroprevalence observed in Group C at 11 weeks is unknown, one plausible explanation is an immunity boost following the second *M. hyopneumoniae* vaccine dose at 6 weeks; however, our data do not directly demonstrate this. Nevertheless, it should be emphasized that serum antibodies are not suitable for evaluating protective immunity after vaccination, since a direct correlation between serum antibody concentration and protection against *M. hyopneumoniae* has never been demonstrated [6,41,42]. By the end of the fattening period, seroprevalence increased strongly in group B (reaching 100% by 25 weeks) and modestly in Group C (reaching 50% by 23 weeks). These seroconversions are indicative of contact with the bacterium by the end of the fattening period, as corroborated by PCR testing in laryngeal swabs. However, no seroconversion was observed in group A, likely due to the later detection of this bacterium in this group (only 2 weeks before slaughter). This observation is also consistent with the lower prevalence of pneumonia lesions observed at slaughter in pigs from Group A. All the aforementioned findings underscore the importance of effective control measures to minimize the impact of *M. hyopneumoniae* on growth performance and economic returns in swine production systems.

In terms of PCVD control, all three vaccination protocols achieved a satisfactory outcome. As agreed by the scientific community, PCVD diagnosis should be based on the following criteria: (1) the presence of clinical signs, (2) the observation of pathological lesions in lymphoid tissue, and (3) the detection of moderate to high viral load [2]. In our study, no poor-doing pigs, wasting or ill-thrift animals were observed. Therefore, the first criterion for excluding PCVD was fulfilled. There was no PCVD-associated mortality recorded; thus, no pathohistological investigation was performed. PCV2 viremia was used as an antemortem indicator of PCVD. However, because there was no unvaccinated group and all pigs in the study were vaccinated against PCV2, viral load in blood remained either undetectable or at very low levels (<0.7 Log10 GE copies/µL) in all three groups. The lack of clinical signs and PCVD-associated mortality, as well as the very low viremia throughout the study, confirmed that all three PCV2 vaccination schemes conferred a successful clinical and virological protection against PCV2. However, there is also the possibility that the PCV2 challenge was absent or very low. Assessing PCV2-specific antibodies by ELISA provides valuable information about the epidemiology of PCV2 and humoral immunity after vaccination, particularly in seronegative herds [43]. However, interpreting serology in clinical herds is more complex. In our study, serological investigation showed that PCV2 antibodies lasted longer in group A (100% seroprevalence at 19 weeks), while in Groups B and C, they declined faster and earlier (from 11 weeks onward). The clinical relevance of this finding is unknown. In addition, Group B experienced an increase in seroconversion prior to slaughter, which is indicative of contact with the virus and active circulation of PCV2. It is important to mention that the test used to evaluate PCV2 antibodies cannot discriminate between antibodies induced by vaccination or those induced by infection [44]. However, as no viremic pigs were detected before 19 weeks of age, the authors assumed that the antibodies detected, at least up to the age of 19 weeks, were likely due to vaccination.

An important limitation of our study was the low infection level with both *M. hyopneumoniae* and PCV2 observed during the trial. Both agents were detected by PCR, confirming their circulation on the farm during the study and suggesting subclinical infections. However, in the absence of an unvaccinated control group and without clinical signs of disease, it is not possible to quantify the level of the challenge and its impact on the differences in performance seen among groups.

With regard to safety, the investigated vaccine showed a good safety profile, which is consistent with previous observations [21]. No serious adverse events, including deaths or anaphylactic reactions, occurred after vaccination. Only two pigs presented very mild reactions, both of which fully recovered within 15 min. These observations are consistent with the performance recorded after vaccination, as no growth retardation was detected during the nursery-growing period (day 0–100).

Nowadays, there is a societal debate about the sustainability of livestock production [45]. In swine production, improved pig health enhances performance, leading to more efficient production—more kilograms of meat produced in a shorter time—reduced resources needed per kilogram of pork produced, and therefore improved overall sustainability [46]. In this context, vaccines have proven to be an economically beneficial alternative to improve the health of pigs infected with both pathogens [2,6]. In our study, we assessed the economic sustainability of vaccination against these two pathogens. Overall, vaccination with the single-injection RTU vaccine not only improved animal health but reduced the number of injections required by two compared with vaccination scheme C. Altogether, this resulted in an improved economic sustainability, with an increased profit of RMB 54–69 per pig sold. In our study, the observed profit is likely a conservative estimate, since individual feed intake could not be measured and potential gains in feed conversion ratio were not included in the economic calculation. Similarly, the number of person-hours spent on vaccination was not recorded and was therefore not included in this calculation. These limitations warrant further research to elucidate the potential benefits of pig vaccination in promoting sustainability.

## 5. Conclusions

This clinical trial demonstrated that an RTU bivalent vaccine targeting PCV2 and *M. hyopneumoniae* infections is both effective and safe under the field conditions studied. The tested vaccine enhanced growth performance and significantly reduced the prevalence of pneumonia lesions, while effectively preventing disease caused by both PCV2 and *M. hyopneumoniae*. Furthermore, the findings confirmed that using an RTU vaccine for these diseases represents a sustainable approach and offers noteworthy economic advantages. This approach supports pig health and welfare, simplifies vaccine administration for farm personnel, and provides a practical foundation for further research into the vaccine’s role in promoting sustainability.

## Figures and Tables

**Table 1:** Performance results. Live weight (kg) and Average Daily Gain (ADG; mean ± SEM; grams/pig/day) in vaccinated pigs calculated during the nursery-growing period (day 0 to day 100), finishing period (day 100 to slaughter), and during the overall period (weaning to slaughter). Days to slaughter, mortality % and runt % calculated for the overall period.

	Group A	Group B	Group C
**Live weight**	*n* = 320	*n* = 309	*n* = 309
at day 0 (mean ± SD)	5.08 ± 0.60	5.07 ± 0.60	5.09 ± 0.60
at day 100 (mean ± SD)	85.73 ± 9.41	80.59 ± 9.55	80.27 ± 8.64
prior to slaughter (mean ± SEM)	145.6 ± 0.8 ^A^	141.6 ± 0.8 ^B^	141.4 ± 0.8 ^B^
Coefficient of variation Live weight prior slaughter	8.71%	9.45%	9.82%
**ADG**			
day 0–100 (mean ± SEM)	796.6 ± 5.3 ^A^	742.9 ± 5.4 ^B^	752.3 ± 5.4 ^B^
day 100—slaughter (mean ± SEM)	1131.3 ± 17.5 ^A^	1160.0 ± 18.1 ^A^	1156.1 ± 18.4 ^A^
day 0—slaughter (mean ± SEM)	911.1 ± 5.0 ^A^	885.1 ± 5.0 ^B^	890.7 ± 5.0 ^B^
**Days to slaughter**	154.2	154.2	153.0
**Mortality %**	5.3 ^A^	4.5 ^A^	5.8 ^A^
**% pigs under slaughter weight**	0.63 ^A^	0.65 ^A^	0.00 ^A^

^A,B^ Different superscripts within the same row show significant differences among groups (*p* < 0.05).

**Table 2:** Lung lesions evaluation. Prevalence of pneumonia and Lung Lesion Score (mean ± standard deviation) of pigs vaccinated with three different schemes (A, B and C).

	Group A (*n* = 63)	Group B (*n* = 67)	Group C (*n* = 65)
Prevalence of Pneumonia	36.5% ^A^	92.5% ^B^	58.5% ^B^
Lung Lesion Score (LLS)	0.92 ± 1.65 ^A^	5.93 ± 4.08 ^B^	1.43 ± 2.06 ^A^
# lungs with			
Score 0	40	5	27
Score 1–4	20	31	34
Score 5–9	3	19	4
Score 10–14	0	11	0
Score 15–19	0	1	0
Score 20–24	0	0	0
Score 25–28	0	0	0

^A,B^ Different superscripts within the same row show significant differences among groups (*p* < 0.05). # refers to the number of lungs scored.

**Table 3:** Serological results. Prevalence of seropositive animals against PCV2, *M. hyopneumoniae* and *A. pleuropneumoniae*.

	Group	7 Week	11 Week	15 Week	19 Week	23 Week	25 Week
PCV2	A	100%	100%	100%	100%	80%	40%
	B	100%	100%	90%	70%	50%	90%
	C	100%	100%	90%	70%	20%	20%
*M. hyopneumoniae* *	A	40%	60%	50%	60%	30%	20%
	B	10%	0%	10%	10%	0%	80%
	C	70%	100%	20%	20%	50%	50%
*A. pleuropneumoniae* *	A	80%	10%	0%	0%	0%	10%
	B	70%	60%	0%	0%	0%	0%
	C	80%	70%	0%	0%	0%	0%

* For *M. hyopneumoniae* and *A. pleuropneumoniae* positive and doubtful results were considered as positive.

**Table 4:** Safety results. Severe, moderate and mild systemic reactions recorded after vaccination.

Systemic Reactions	Group A	Group B	Group C
Severe	0/320	0/309	0/309
Moderate	0/320	0/309	0/309
Mild	2/320	2/309	0/309
Local reactions	0/320	0/309	0/309

**Table 5:** Economic assessment. Revenue, costs and net profit (in RMB) of pigs vaccinated from Group A (tested vaccine, used as reference) compared with Groups B and C.

Economic Parameters	Group A	Group B	Δ (A–B)	Group C	Δ (A-C)
Average Final Live Weight (kg)	145.55	141.52	4.03	141.45	4.10
Prevalence of healthy lungs (%)	64.50	7.50	57.00	41.50	23.00
# needles used/vaccinated pig	1	1	0	3	−2
Mortality %	5.30	4.50	0.80	5.80	−0.50
**Revenue (RMB)**					
Per Pig/sold ^1^	2196.35	215.54	60.81	2134.48	61.87
Per healthy lung sold ^2^	1.29	0.15	1.14	0.83	0.46
**Costs (RMB)**					
Needle costs/pig sold ^3^	1.06	1.05	0.01	3.19	−2.13
Cost of Mortality ^4^	51.30	43.56	7.74	56.14	−4.84
**Net profit (RMB)**					
Per pig sold	2145.28	2091.08	54.20	2075.98	69.30

# refers to number of needles; ^1^ Average price of one kilogram of a live pig sold at the abattoir: RMB 15.09/kg live weight; ^2^ Average price of a healthy lung sold at the abattoir: RMB 2.00/lung; ^3^ Price of a needle: RMB 1.00/unit; ^4^ Cost of mortality: RMB 9.68/pig placed for each incremental improvement of 1%.

## Data Availability

The data presented in this study are contained within this article.

## Abbreviations

The following abbreviations are used in this manuscript:

RTU	Ready-To-Use
PCV2	Porcine Circovirus Type 2
*M. hyopneumoniae*	*Mycoplasma hyopneumoniae*
ADG	Average Daily Gain
PCVD	Porcine Circovirus Disease
PRRSV	Porcine Reproductive and Respiratory Syndrome Virus
PCR	Polymerase Chain Reaction
ORF	Open Reading Frame
LLS	Lung Lesion Score
SPES	Slaughterhouse Pleurisy Evaluation System method
Real-time PCR	Real-time Polymerase Chain Reaction
AUC	Area Under the Curve
ELISA	Enzyme-Linked Immunosorbent Assay
RMB	Renminbi
AN(C)OVA	Analysis of Covariance
SEM	Standard Error of the Mean
GE	Genomic Equivalent
*A. pleuropneumoniae*	*Actinobacillus pleuropneumoniae*
***Δ***	Difference
VLPs	Virus-Like Particles
